# Increasing health facility deliveries in Cambodia and its influence on child health

**DOI:** 10.1186/s12939-019-0964-8

**Published:** 2019-05-14

**Authors:** Hayley Pierce

**Affiliations:** Brigham Young University, 2036 JFSB, Provo, Ut 86402 USA

**Keywords:** Cambodia, Maternal health, Child health, Health inequality, Increasing Health Facility Deliveries in Cambodia and its influence on Child Well-Being

## Abstract

**Background:**

A growing number of women in Cambodia are seeking support from health facilities during delivery, up from 8% in 2000 to 82% in 2014. This growth may be attributed to increased national level attention to incentivize hospital births and reduce potential barriers. This paper address three related questions regarding the impact of increased utilization of health care in Cambodia. First, did increasing health facility deliveries occur most among disadvantaged women? Second, as health facility utilization increased, did the benefit of delivery location on child health outcomes weaken? Finally, did socioeconomic disparities in child outcomes decline as a result of increased health facility deliveries?

**Methods:**

Data is from the 2010 and 2014 Cambodian Demographic and health surveys. Regression models include logistic regression to predict utilization of a health facility, linear regression to predict child nutritional status and Cox regression to measure child survival. Propensity score matching was used to account for selectivity.

**Results:**

Analysis shows that health facility delivery is associated with better nutritional status and survival and the effectiveness of a health center delivery remains with this rapidly increasing care. However, the largest increases in delivery at a health facility did not occur among less educated, less wealthy, and rural Cambodian women, and inequalities in child health outcomes remain.

**Conclusions:**

Cambodian women have participated in a rapid increase in health center deliveries and those health facility deliveries remain beneficial for future child outcomes. However, initiatives to increase care are not addressing inequity in access to care among disadvantaged women. Additionally, disparities in children’s health outcomes remain, suggesting that health facility births are not sufficient in reducing disparities among children of disadvantaged mothers. Moving forward, current initiatives are rapidly increasing facility deliveries and maintaining their efficacy, but further efforts need to be placed on targeting disadvantaged women and their children.

## Background

Delivering a child at a health facility is known to have many health benefits, especially in developing countries where this care is often limited or underutilized [1–4]. Inadequate utilization of healthcare is further intensified by socioeconomic inequalities such that disadvantaged mothers and children are placed at further disadvantage through poor health outcomes [5–8]. Delivery at a health facility does not have implications for the mother alone; the health of her child is directly and indirectly tied to delivery location. Directly, children born in a health facility receive crucial care at birth. Indirectly, mothers who deliver at a health facility tend to build trust with the health care system which increases future health service utilization for her child. Those services include, but are not limited to: immunizations, oral rehydration, well-baby checkups, preventive care, malaria treatment, and other future care for her child [9, 10]. Annually worldwide, about 300,000 women die as a result of pregnancy or childbirth, as do 9 million children younger than 5 years [11]; about 18,000 of those maternal deaths and 400,000 of those child deaths are in southeast Asia. Although maternal and child mortality are on the decline in southeast Asia, there are still significant disparities, and greater equity is the key to further mortality reduction [12].

This paper focuses on Cambodia, one southeast Asian country that has made drastic gains in the percentage of women who deliver children at a health facility. An extensive portion of the health literature identifies social factors, such as education, residence and wealth, at the root of health inequalities. This relationship naturally makes these social factors an important site for interventions to cause changes in health [13–15]. The aim of this paper is to address three related questions regarding the impact of increased utilization of health care in Cambodia. First, did increasing health facility deliveries occur most among disadvantaged women? Second, as health facility utilization increased, did the benefit of delivery location on child health outcomes weaken? Finally, did socioeconomic disparities in child outcomes decline as a result of increased health facility deliveries?

### Cambodian context

Noting similarities and differences between bordering countries within southeast Asia is useful to determine health care progress and deficiencies. Of the 177 countries listed in the United Nations Human Development Index, Cambodia ranks 129, Myanmar 130 and the Lao People’s Democratic Republic 133, while Thailand ranks 74. This index is based on a country’s life expectancy at birth, expected years of schooling and standard of living. In 2015, the maternal mortality ratio (MMR), which is the annual number of female deaths per 100,000 live births from any cause related to or aggravated by pregnancy, within these select southeast Asian countries range from a low of 20 in Thailand to highs of 161 in Cambodia, 197 in Laos, and 178 in Myanmar [16]. Furthermore, infant mortality rates (IMR) per 1000 live births in these countries are 11 in Thailand, 30 in Cambodia, 46 in Myanmar, and 47 in Laos. For reference, the World Bank suggests the MMR in the United States in year 2015 was 14 deaths per 100,000 live births while the IMR was 5.7. This vast discrepancy between maternal and child health in Thailand and its neighboring countries suggest that improvements are possible within the region. Recent health care expansions and initiatives in Cambodia may create a realistic plan by which other neighbors to Thailand can continue to lessen the gap in maternal and child health outcomes.

Recent policy initiatives in Cambodia include improving supply side barriers [17], integration of traditional birth attendants into the health care system through trainings and job placements [18], and the introduction of the Government Midwifery Incentive Scheme in 2007. This initiative is a financing scheme which motivates skilled birth attendants to promote deliveries in public health facilities through cash and salary incentives [19]. The Royal Government of Cambodia also renewed its Emergency Obstetric & Newborn Care Improvement Plan which increased Obstetric facilities from 25 to 37 and Newborn Care facilities from 19 to 110 [20]. Since 2000, the Royal Government has also increased attention and training for midwives by ensuring all health centers have at least one primary midwife and incentivizing hospital births through improved service quality. Simultaneously, the government has banned deliveries by unintegrated and untrained traditional birth attendants and strongly discourages home births. The Government has also worked to reduce health care costs among the poor by introducing health equity funds. These funds provide health insurance for the poor by reimbursing health providers for free services provided to individuals identified as poor. Since the introduction of these funds in 2000, over 2.5 million poor people in Cambodia have access to these services [20, 21]. Eligible households can access subsidized or free health care, including maternal services. However, limitations of these health initiatives exist, making it unclear to what extent improvements in health can be attributed to them. Many of these initiatives focus on curative care, opposed to preventative care, and many of these services have not been implemented at the national scale. While all of these initiatives may reasonably have contributed to improved health care and health outcomes, the extent to which this is the case is unclear [17] .

A growing number of women in Cambodia are seeking support from health facilities during delivery, up from 8% in 2000 to 82% in 2014. Equally encouraging, improved obstetric care for women and involvement with a health facility through promoted breastfeeding and vaccinations have helped prevent newborn and early child deaths [22]. Despite dramatic mortality reductions over recent years, inequities in access to health care are preventing all population groups in the country from seeing health gains with this increasing health care access. Pregnant women do not always receive sufficient care needed during their deliveries, and the quantity and quality of care often varies by sociodemographic factors such as residence, wealth and education [6, 8, 14, 23, 24]. To further complicate the matter, more access to health services does not necessarily promote more equality in treatment and outcomes. If all women are able to deliver at a health facility, the quality and benefits of that care may still be impacted by a woman’s sociodemographic circumstance; such that socioeconomic status embodies a spectrum of resources that protect or harm health no matter what health services are accessed [25].

An increasing number of studies have looked into rising maternal health care in Cambodia [17, 19, 26–30], though many have not looked at the effects of socioeconomic disadvantage and child health. This paper focuses on the last 4 years of improved health care in Cambodia, when utilization of health facilities surged above 50% for the most disadvantaged women. The research questions and methods draw upon work looking at a similar circumstance in Rwanda [9]. Both Cambodia and Rwanda experienced genocides and periods of political and social turmoil, but have recently devoted governmental policy and funding to improve maternal health care. As a result, both countries experienced impressive gains in health facility deliveries, providing a roadmap to health care progress for their neighboring countries.

Acknowledging the vast growth in facility utilization for child delivery, and the importance of social factors for health outcomes [13–15]; this paper seeks to understand how this increased utilization interacts with socioeconomic disparities among women and how that interaction influences child well-being. To understand these dynamics, this paper addresses three associated questions concerning the impact of increased use of health facility deliveries in Cambodia, then situates these findings within national health care initiatives. First, I assess the change in the influence of urban residence, wealth and maternal education on delivery at a health facility. Second, I assess whether there is a change in the child wellbeing benefits associated with delivery at a facility. Finally, I assess whether there is increased equality in child wellbeing, measured by survival and nutrition. The analytical framework will proceed as follows: first, logistic regression is used to predict utilization of a health facility. Second, linear regression is used to predict child nutritional status. Next, Cox regression is used to measure child survival. Lastly, in order to account for selectivity in health facility deliveries, propensity score matching is used.

## Methods

Data for this analysis is taken from Cambodian Demographic and Health Surveys (DHS) conducted in 2010 and 2014. The DHS are nationally representative samples of women of childbearing age conducted in over 90 countries. The survey is conducted by trained field staff, field editors, and team leaders from the implementing agencies. In the case of Cambodia, the project implementation agencies include: The Directorate General for Health of the Ministry of Health and the National Institute of Statistics of the Ministry of Planning. Extensive steps are taken by survey administers to ensure the data are comparable across countries and properly reflect the situations and people they intend to describe. I have combined the surveys to examine change over time based on a total sample of 15,397 cases, which is inclusive of all observations with missing values. The sample sizes for each year are: 2010 (*n* = 8232) and 2014 (*n* = 7165). The outcome variables include delivery at a health facility, child survival, and child nutritional status measured at the time of the survey. Nutritional status is measured by the child’s height for age compared to the World Health Organization standardized model. Child survival is based on whether the child is still living and the duration from birth to death or date of the survey. Delivery at a health center is a dichotomous variable comparing women who delivered in a public or private health facility and women who delivered in a home (either theirs or others). Simply as a note, among only the women who delivered in a health facility in 2010, 80.8% of births were in a public facility compared to 83.1% of births in 2014, suggesting there was no major shift from public to private facilities over this time period.

Included in the analysis were indicators of inequality including education, wealth and urban residence. A woman’s self-reported educational attainment was measured as “no education,” “incomplete primary,” “complete primary,” “incomplete secondary,” and “complete secondary and higher.” It is important to note that a growing number of women are getting education over this time period, no education is down from 22% in 2010 to 14.4% in 2014. Inversely, incomplete secondary and complete secondary is up from 21% in 2010 to 28% in 2014, and 4% in 2010 to 8% in 2014, respectively. Household wealth is a wealth index that places individual households on a standardized scale of relative wealth including assets, housing materials, and access to water and sanitation. Finally, residence is a dichotomous variable comparing women who live in urban areas and women who live in rural areas. The proportion of urban women remained consistent across the two time periods at 25.7% urban in 2010 and 27% urban in 2014. The DHS decides the difference between urban and rural areas in terms of living standards which tends to be the degree of population concentration or density [31]. Other criteria may also be considered, including the ease of access to healthcare, schools, or transportation, the percentage of the population involved in agriculture or the availability of electricity or piped water.

Control variables were selected based on a what was available in the DHS and a literature search of the most influential factors determining use of health facility and child outcomes. First birth is a dichotomous variable for children who were the first birth to the woman. Maternal medical knowledge is measured by the mother’s understanding of her ovulation cycle. The DHS routinely asks women when in her ovulation cycle she is most likely to get pregnant, with the options being during her period, after period ended, middle of the cycle, before period begins, at any time, or do not know. I recoded the variable 1 if the woman reports she is most fertile during the middle of her ovulation cycle, and 0 if she answered otherwise. Birth interval is a continuous variable from the date of the birth of interest to the date of the previous end of pregnancy or birth. Marital status is a dichotomous variable coded 1 for women with no prior marriage or a disrupted marriage through death or divorce compared to currently married women. And finally, mothers age is a continuous variable based on reported age in years.

Logistic regression analysis was performed to determine the odds of delivery at a health facility in order to see if more disadvantaged women were receiving care. Year is coded 0 for 2010 and 1 for 2014 so the coefficients for education, wealth and residence show the degree of social inequality in 2010. The interactions between year and education, wealth, and residence show how much the coefficients changed by 2014. To address the second question, I add delivery at a health facility as an independent variable and use linear regression to predict child nutritional status and Cox regression to predict child mortality. I also add the interaction between health facility and year to show change by 2014.

The third question is addressed by including social factors and interactions with year in the models predicting child health and survival. In order to account for the selectivity process that occurs when selecting a delivery location, I use a propensity score matching (PSM) approach. The motivation for and explanation of propensity score matching has been described in detail elsewhere [9]. Briefly, PSM is able to create “treatment” and “control” groups based on characteristics that influence delivery location. Those include: first birth, maternal medical knowledge, marital status, previous child death, and maternal age. Individuals with the same distribution of listed covariates are given a propensity score, then randomly assigned into the treatment and control groups, just as randomization occurs in experimental studies. The characteristics included in the model were available measures that prior research has found to be frequently and consistently related to delivery location [1–3, 5, 7, 10, 12, 32]. The propensity score prediction is only done for those observations that do not have missing values for any of the covariates. Missing values were treated in a variety of ways with no significant changes to the findings. I then use a weighted regression approach where the weights are derived from the propensity score in order to reduce selection bias. For subjects in the treatment group, this weight is equal to 1/*p*_*i*_ and for subjects in the control group the weight is equal to 1/(1 − *p*_*i*_) [33] . I imposed a nearest neighbor condition, matching 1 treated unit with 5 controls, but no replacement model was specified. This process yielded a set of matching weights for the control group, which allows for an appropriate set of counterfactual cases. Stata 15.1 was used for the analysis. Each model was estimated with and without the propensity score weight in order to understand the role of selectivity on the child health outcomes.

## Results

### Question 1: did increasing health facility deliveries occur most among disadvantaged women?

**Fig. 1:**
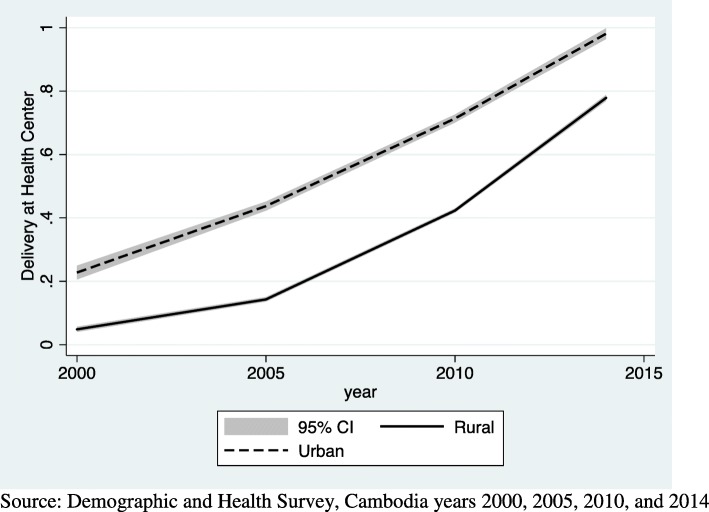
top Line graph of urban and rural residence and delivery at a health facility by year. Source: Demographic and Health Survey, Cambodia years 2000, 2005, 2010, and 2014

**Fig. 2:**
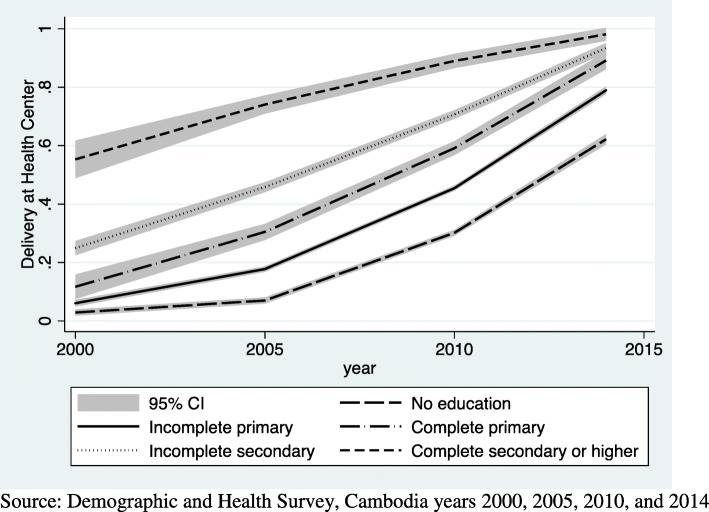
top Line graph of educational attainment and delivery at a health facility by year. Source: Demographic and Health Survey, Cambodia years 2000, 2005, 2010, and 2014

**Fig. 3:**
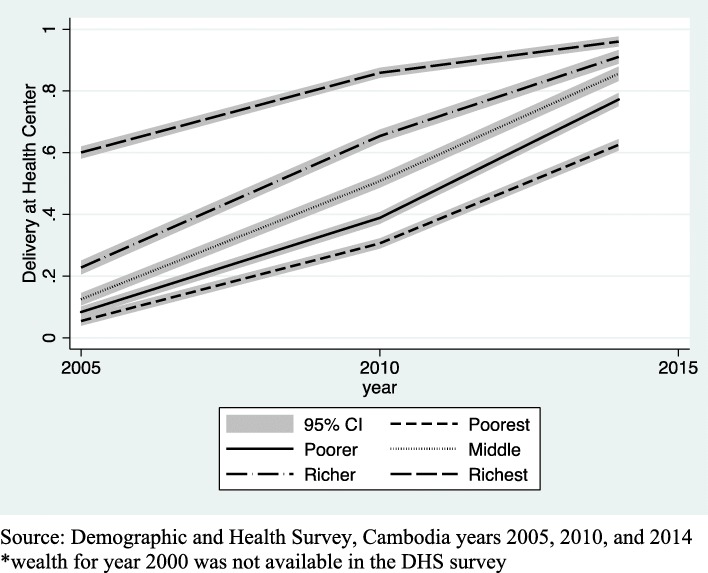
top Line graph of household wealth and delivery at a health facility *. Source: Demographic and Health Survey, Cambodia years 2005, 2010, and 2014. *wealth for year 2000 was not available in the DHS survey

Figures 1, 2 and 3 use DHS data to show the growth in health facility deliveries according to each sociodemographic factor for the years 2000 to 2014. Each graph is presented with a 95% confidence interval (CI). Analysis for this paper is focused on growth since 2010, however, seeing the trend since 2000 is useful to see the overall growth. Fig. 1 shows the increase in utilization of health facilities for delivery among urban and rural women in Cambodia. Use increased among both urban and rural women, but the increase was slightly steeper for rural women since 2010. Fig. 2 shows the increasing use of health facilities for delivery according to educational attainment. Gains were notable in all but the highest educational group, with the most rapid increases taking place since 2010. And finally, Fig. 3 shows results for wealth. Similar to education, increases have accelerated since 2010 for all but the wealthiest group. These graphs show the overall trend in health facility deliveries over this time period, not the statistical significance of the relationship.

Table 1 shows the results of the logistic regression used to estimate the propensity score of the likelihood of utilizing a health facility for delivery. Simultaneously, this table shows differences in the type of women who deliver in health facilities. In 2010, the odds that women delivered in a health facility increased by 1.30 with each step up in educational attainment (i.e. incomplete primary to complete primary) and by 1.42 times with each one standard deviation change in wealth. Additionally, the odds of facility delivery were over 2 times higher in urban areas than in rural areas. By 2014, the odds of urban women delivering in a health facility were slightly, though not significantly reduced while the odds of facility delivery were slightly increased for the wealthiest and most educated women. The relative odds of delivering in a health center in 2014 equals the product of the coefficient times the interaction term. By 2014 the odds of facility delivery among educated women would increase by 1.421 (1.301*1.092) with each step up in education. The interactions for wealth, and urban are near unity and not significant, while the interaction with education is slightly elevated, suggesting that the influence of each did not change much over time. All-together, wealthier, more educated, and urban living women have increased odds of utilizing a health facility for delivery in both 2010 and 2014, with only minimal changes between the two time periods. This suggests that yes, health facility deliveries are increasing in Cambodia, but the increased care did not occur most among disadvantaged women. This occurred while controlling for birth interval, first birth, maternal medical knowledge, marital status, previous child death and mothers age.

**Table 1:** Logistic regression predicting utilization of a health facility (*N* = 15,397)

	Odds ratio	s.e.
Indicators of Inequality		
Maternal education	1.301**	0.034
Household wealth	1.422**	0.031
Urban residence	2.068**	0.147
Year	1.092**	0.315
Interactions with year and:		
Maternal education	1.092*	0.047
Household wealth	1.061	0.040
Urban residence	0.857	0.120
Controls		
Birth interval	1.006**	0.001
First birth	1.588**	0.081
Maternal medical knowledge	1.396**	0.087
Never married/disrupted marriage	0.910	0.085
Previous child death	0.706**	0.039
Mothers age	0.988*	0.004

**p < 0.001 *p < 0.05

### Question 2: as health facility utilization increased, did the benefit of delivery location on child health outcomes weaken?

Tables 2, 3, 4 and 5 show the difference in child nutritional (Tables 2 and 3) and survival outcomes (Tables 4 and 5) for both the unmatched and matched sample. The analysis of the unmatched sample (Tables 2 and 4) shows that child health status does improve if the child is delivered at a health center. Children are one-half of a standard deviation higher on height for age and have mortality rates over 65% lower than non-health facility born counterparts. This supposition is challenging due to the selectivity that occurs when selecting a delivery location. Use of a health facility may be selective of more advantage women with resources to access a health facility, or it could indicate women with higher risk pregnancies. To understand the influence of selectivity on effects of delivery at a health center, I use propensity score matching.

**Table 2:** Regression predicting child nutrition with presence of a health facility (HF) and measures of socioeconomic inequality for unmatched sample

	Only HF	s.e.	Without HF	s.e.	With HF	s.e.
Health Facility	51.810**	3.128			27.644**	4.663
Indicators of Inequality						
Maternal education			6.180**	4.612	6.467*	2.171
Household wealth			13.423**	1.917	11.145**	1.951
Urban residence			4.936	5.807	1.595	5.818
Year			19.037*	6.632	12.627	7.355
Interactions with year and:						
Maternal education			−3.666	2.717	−2.807	2.738
Household wealth			2.119	2.567	3.397	2.609
Urban residence			−4.434	7.883	−1.568	7.881
Health facility					−8.666	7.186
Controls						
Birth interval			.444**	.065	0.409**	0.065
First birth			−7.611*	3.488	−9.453*	3.489
Maternal medical knowledge			10.994*	3.595	10.102*	3.587
Never married/disrupted marriage			−15.675*	6.648	−16.168*	6.630
Mothers age			−2.097**	.282	−1.998**	0.281

*N* = 7972

**p < 0.001 *p < 0.05

**Table 3:** Regression predicting child nutrition with presence of a health facility (HF) and measures of socioeconomic inequality for matched sample

	Only HF	s.e.	Without HF	s.e.	With HF	s.e.
Health facility	29.578**	3.993			26.748**	5.180
Indicators of Inequality						
Maternal education			3.450	3.279	4.044	3.262
Household wealth			10.796**	2.621	10.628**	2.630
Urban residence			−4.371	7.522	−5.026	7.520
Year			4.984	8.980	−3.052	9.740
Interactions with year and:						
Maternal education			1.339	4.028	0.771	3.973
Household wealth			4.199	3.327	3.570	3.377
Urban residence			−0.213	10.436	−0.542	10.413
Health facility					−2.552	10.122
Controls						
Birth interval			.360**	.086	0.371**	0.085
First birth			−11.256*	4.713	−11.465*	4.734
Maternal medical knowledge			4.984	4.987	5.715	4.957
Never married/disrupted marriage			−18.021	9.887	−15.645	9.772
Mothers age			−2.063**	.388	−2.117**	0.386

*N* = 6733

**p < 0.001 *p < 0.05

**Table 4:** Cox regression predicting child survival with presence of a health facility (HF) and measures of socioeconomic inequality for unmatched sample

	Only HF	s.e.	Without HF	s.e.	With HF	s.e.
Health facility	0.345**	0.041			0.602**	0.101
Indicators of Inequality						
Maternal education			0.825*	.069	0.848*	0.071
Household wealth			0.826*	.055	0.862*	0.058
Urban residence			0.752	0.182	0.806	0.195
Year			0.750	0.194	0.912	0.221
Interactions with year and:						
Maternal education			0.931	0.133	0.917	0.132
Household wealth			0.909	0.110	0.889	0.110
Urban residence			0.924	0.428	0.874	0.406
Health facility					1.257	0.101
Controls						
Birth interval			0.987**	0.003	0.989**	0.003
First birth			1.394*	.214	1.480*	0.231
Maternal medical knowledge			0.584*	.122	0.601*	0.125
Never married/disrupted marriage			1.103	.284	1.109	0.286
Mothers age			1.056**	.010	1.056**	0.010

*N* = 7972

**p < 0.001 *p < 0.05

**Table 5:** Cox regression predicting child survival with presence of a health facility (HF) and measures of socioeconomic inequality for matched sample

	Only HF	s.e.	Without HF	s.e.	With HF	s.e.
Health facility	0.623*	0.095			0.674*	0.124
Indicators of Inequality						
Maternal education			0.790	0.103	0.785*	0.101
Household wealth			0.862	0.071	0.866	0.072
Urban residence			0.494*	0.165	0.500*	0.165
Year			0.722	0.223	0.689	0.262
Interactions with year and:						
Maternal education			0.933	0.186	0.940	0.188
Household wealth			0.866	0.106	0.867	0.108
Urban residence			1.719	0.888	1.706	0.882
Health facility					1.308	0.469
Controls						
Birth interval			0.990*	0.003	0.990*	0.003
First birth			1.422	.258	1.434*	0.263
Maternal medical knowledge			0.655	.226	0.654	0.225
Never married/disrupted marriage			1.538	.634	1.557	0.643
Mothers age			1.063**	.014	1.064**	0.014

*N* = 6733

**p < 0.001 *p < 0.05

In the matched sample (Tables 3 and 5), children born in a health center score .29 standard deviations higher on nutritional status and have mortality rates about 40% lower, compared to children born in a home. These differences are not greatly altered when interactions and controls are added. The coefficients and the interaction terms for the indicators of inequality are similar in the matched and unmatched samples suggesting that the use of propensity score matching to account for selectivity does not modify my conclusions.

### Question 3: did socioeconomic disparities in child outcomes decline as a result of increased health facility deliveries?

Results from the matched samples show inequality in health outcomes associated with education, wealth, and residence. For each step increase in educational attainment, a child is 0.04 standard deviations higher in height for age. Similarly, a one standard deviation increase in wealth is associated with a 0.1 standard deviation increase in nutritional status, while urban residence is near unity (Table 3). Looking at survival, household wealth, mother’s education, and urban residence, each are associated with lower rates of child mortality (Table 5). Across all models, the interactions between the indicators of inequality and year are not significant, suggesting that there was not a reduction in these socioeconomic disparities in children’s health outcomes between 2010 and 2014. In the matched models for child health outcomes there was even an increase in disparities associated with maternal education and wealth for nutritional status, and residence for child survival. Similarly, coefficients for the indicators of inequality are similar with and without health facility suggesting that socioeconomic inequality was not mediated by use of a health center.

## Discussion

The aim of the study was to explore the relationship between maternal care at child birth and the future health and survival of that child. Cambodia has made significant progress in improving health care for its women and children by dramatically increasing health facility births since 2000. The final goal is to explore how this increase in utilization is situated within governmental policy and outside interventions to determine how the programs may contribute to maternal care and future child outcomes.

Any discussion of these links should be considered speculative; it is not possible to make causal inferences about the timing of health initiatives and the improvements in health utilization and health outcomes in the available data. It is also possible that initiatives outside the health sector, such as the expansion of quantity and quality of education played a role here [34]. However, it is important to note that these risings rates of facility deliveries have occurred over the same period of time as the health initiatives discussed. In support of discussing these links, the ecological model of health behavior notes that healthy behaviors are thought to be maximized when environments and policies support these healthy choices [35]. The results of the current study provide some evidence of the positive contributions of these initiatives and presents some deficiencies that need to be addressed to produce further progress.

This included the following contributes and deficiencies, respectively:

1. Considering rapid growth in health care among Cambodian women, the efficacy of facility delivery on child nutritional status and survival remains, and even increases over this period.
2. Efforts to increase care are not addressing inequity in access to care among disadvantaged women and disparities in children’s health outcomes remain. A discussion of these results follows.

### Efficacy of facility delivery on child outcomes

The results of this research have shown that the delivery location of a mother has positive implications for child nutrition and survival, and those results remain and even increase over this period of immense growth in health facility utilization. Efforts to improve maternal outcomes in developing countries have focused primarily on training skilled birth attendants and upgrading health care facilities [1, 32, 36–39]. This increase in demand is often met with poorly trained staff and insufficient supplies, though these deficiencies can be overcome through proper training and intervention [40, 41]. Cambodian health care initiatives included a focus on training midwives and incentivizing improved care of patients. Simultaneously, the government aimed to decrease supply side barriers such as limited medicine and birthing supplies [17] . This research suggests that scaling up care, in combination with improving supply side and demand side factors, is able to maintain the benefits of health facility utilization.

### Inequity in access and child outcomes

This research suggests that health care deliveries are increasing, but that increase is not pacing significantly faster for disadvantaged women. A study by Dingle et al. (2013) points out an imperative relationship when they say, “service coverage and equity in service use are interrelated concepts (pg. 8).” It is a process where high levels of coverage reflect low inequity in service use, regardless of socioeconomic advantage and disadvantage. While low coverage across all socioeconomic groups *also* produces low inequity in service use because all people have limited access. Therefore, it is important to note that in Cambodia since 2000, overall coverage has drastically increased among all individuals. However, this research suggests that since 2010 improvements in equity have stalled. This intimates that overall improvements in coverage of services is not purely a consequence of increases in utilization by the wealthiest. However, efforts to reach the most severely disadvantaged women that were not already reached by previous interventions in the early 2000s needs to be the focus moving forward. This will ensure that progress among the most disadvantaged women and children keeps pace with their socioeconomically advantaged counterparts. Similarly, the children of advantaged women continue to see improved nutritional and survival outcomes. This suggests that the location of delivery is not sufficient to reduce inequity in child health, and further policy must address this discrepancy.

Many policy initiatives have focused on equity in maternal health. Health equity funds provide health insurance for the poor, but out of pocket health payments remain high in Cambodia, creating a poverty trap for the poor [17]. Perhaps expense of health care is halting a faster pace in health care utilization among the disadvantaged. Additionally, a qualitative study by Matsuoka and colleagues [42] suggests that non-use of maternal health services among rural women was because of financial barriers, physical barriers, cognitive barriers, organizational barriers, and sociocultural barriers. In further detail, these barriers include cultural beliefs in traditional medicine, a misunderstanding of government financing schemes and how to utilize them, poor access to services, and decreased trust in the quality of formalized health services. These deficiencies may suggest areas of improvement in the coming years.

## Conclusion

The Cambodian government and organizations alike have made a focused effort to address maternal and child health, simultaneously, there have been general improvements in socioeconomic conditions that may have influenced health related behavior for Cambodian families [17]. The results of the current study provide some evidence of the positive contributions of these initiatives and presents some deficiencies that need to be addressed. Cambodian women have participated in a rapid increase in health center deliveries and those health facility deliveries remain beneficial for future child outcomes. However, the pace of increased utilization among disadvantaged women is lower than their advantaged counterparts, suggesting that initiatives to increase care are not addressing inequity in access to care among disadvantaged women. Additionally, disparities in children’s health outcomes remain, suggesting that health facility births are not sufficient in reducing disparities among children of disadvantaged mothers.

Moving forward, other countries, especially those neighboring Cambodia, may benefit from incorporating key features of these initiatives into their national health plans because it is rapidly increasing facility deliveries and maintaining their efficacy. From the review of recent Cambodian policy initiatives, select initiatives may be: increasing the number of obstetric facilities and improving midwife training [20], addressing supply side barriers [17], integrating traditional birth attendants [18], financially incentivizing hospital births for both midwives and pregnant women [19], and providing government level subsidies to health providers that provide free services to poor women [21, 43, 44]. While all of these initiatives may reasonably contribute to rising health facility deliveries and child health outcomes, the extent to which this is the case is unclear [17]. However, in Cambodia and in its neighboring countries, further efforts need to be placed on targeting disadvantaged women. Inequality is a key challenge that must be addressed to ensure that progress continues and that all women and children, no matter their socioeconomic status, see health gains.

## Abbreviations

CI: Confidence interval
DHS: Demographic and Health Survey
HF: Health facility
